# Gender, Season and Management Affect Fecal Glucocorticoid Metabolite Concentrations in Captive Goral (*Naemorhedus griseus)* in Thailand

**DOI:** 10.1371/journal.pone.0091633

**Published:** 2014-03-17

**Authors:** Jaruwan Khonmee, Janine L. Brown, Suvichai Rojanasthien, Anurut Aunsusin, Dissakul Thumasanukul, Adisorn Kongphoemphun, Boripat Siriaroonrat, Wanlaya Tipkantha, Veerasak Punyapornwithaya, Chatchote Thitaram

**Affiliations:** 1 Faculty of Veterinary Medicine, Chiang Mai University, Chiang Mai, Thailand; 2 Center for Species Survival, Smithsonian Conservation Biology Institute, Front Royal, Virginia, United States of America; 3 Chiang Mai Night Safari, Chiang Mai, Thailand; 4 Omkoi Wildlife Sanctuary, Department of National Park, Wildlife and Plant Conservation, Chiang Mai, Thailand; 5 Conservation Research and Education Division, Zoological Park Organization, Bangkok, Thailand; University of Missouri, United States of America

## Abstract

Chinese goral (*Naemorhedus griseus*) are a threatened species in Thailand and the focus of captive breeding for possible reintroduction. However, little is known of their biology or what factors in the captive environment affect welfare. Our objective was to determine the impact of gender, season, and management on goral adrenal activity. We hypothesized that differences in fecal glucocorticoid concentrations would be related to animal density. Fecal samples were collected 3 days/week for 1 year from 63 individuals (n = 32 males, 31 females) at two facilities that house the majority of goral in Thailand: Omkoi Wildlife Sanctuary (Omkoi), an off-exhibit breeding center that houses goral in individual pens (16 pens; n = 8 males, 8 females) and in small family groups (8 pens; n = 8 males, 8 females); and the Chiang Mai Night Safari (NS), a zoo that maintains 31 goral (n = 17 males, 14 females) in one large pen. Glucocorticoid metabolite concentrations were higher in male than female goral at Omkoi throughout the year, and there was a seasonal effect on adrenal activity (*p*<0.05). Goral at Omkoi and NS were used to test the effect of animal density on fecal glucocorticoid excretion of goral housed in similar-sized enclosures. Overall, the highest levels were found at NS (n = 31 adults/pen; 27 m^2^ per animal) compared to Omkoi (n = 2 adults/pen; 400 m^2^ per animal) (*p*<0.05). Overall findings support our hypothesis that animal density and aspects of the captive environment impact adrenal steroid activity in captive goral. In addition, gender and season also had significant effects on glucocorticoid metabolite production. Potential stressors pertaining to the welfare of this species were identified, which will guide future efforts to improve management and create self-sustaining and healthy populations of this threatened species.

## Introduction

Chinese, or grey long-tailed, goral (*Naemorhedus griseus*) are small ungulates with bovid-like features that inhabit mountainous areas of Myanmar, China, India, Thailand, and Vietnam [1]–[3]. They are agile and easily traverse steep cliffs and rocky crags [3]. Goral are diurnal and live in small family groups of 4–12 individuals; males are territorial and defend home ranges of 25–40 hectares in size [3]. Goral were listed in 1992 as one of 15 protected species under the Wild Animal Reservation and Protection of Thailand [4], and are categorized as Vulnerable by the IUCN Red List [3]. Most goral in Thailand are found within seven protected areas in the northern part of the country, restricted to hills along the Ping River, in the Chiang Mai, Mae Hong Son and Tak Provinces [3], [4]. There has been no estimate of the total population size of wild goral, but numbers are declining throughout their range because of habitat loss, over-hunting and disease [3], [4]. As a result, there is increasing interest by the Zoological Parks Organization of Thailand to initiate captive breeding programs for goral reintroduction. Today, captive populations are viewed as important “insurance” against environmental or anthropomorphic catastrophe [5], [6], so efforts to improve breeding management for species like the goral are warranted.

There are about 100 goral housed among three captive facilities in Thailand, Omkoi Wildlife Sanctuary (Omkoi), Chiang Mai Night Safari (NS) and Chiang Mai Zoo. Omkoi and NS hold all but three of Thailand's captive goral and although there is breeding at both facilities, the populations are not self-sustaining. Goral management at the two facilities differs significantly. Omkoi is not open to the public and houses over 60 goral in small breeding groups (male, female and offspring) in both large- and small-sized enclosures, whereas NS is a tourist attraction and has 31 goral (n = 17 males, 14 females) kept together in one large enclosure. Given that wild goral live in small family groups (herds of four to 12 individuals, usually with one breeding male) within established territories [3], this study examined how these differences in captive housing conditions in Thailand impact individual animal welfare through assessment of adrenal function. There are no published hormonal data, reproductive or adrenal, for goral of either sex. So, there is a need to more fully characterize the biology of this species and identify factors that affect reproduction and welfare to aid propagation and conservation efforts and guide management strategies.

A number of potential stressors exist in captive environments and animal responses can be species-specific. Studies have shown that stress from inadequate housing conditions, inappropriate social interactions or other husbandry factors can lead to heightened glucocorticoid production [7]–[18]. If a stressor persists or causes consecutive stress responses, chronic glucocorticoid exposure can lead to a number of problems, including abnormal animal behavior, decreased libido, suppressed immune function, poor population performance, and disruption of reproductive hormone secretion [19]–[23]. One way to monitor welfare is through the analysis of hormonal metabolites excreted in urine and feces [24], [25]. Non-invasive glucocorticoid metabolite monitoring is now well established as a valuable tool for understanding adrenal function, and offers significant advantages over blood sampling for assessing stress status [13], [22]. In particular, fecal glucocorticoid metabolite analysis techniques have been developed for a number of domestic and wildlife species, which have led to improved *ex situ* management [12], [23], [26], [27]. The ease of fecal collection without animal disturbance and that data reflect pooled values over time makes this a particularly attractive approach for zoo-held species [22], [23].

The objective of this study was to use fecal glucocorticoid analyses to determine the influence of gender, season and management on metabolite concentrations in male and female goral. Based on the natural history of goral, we tested the hypotheses that lower fecal glucocorticoid concentrations would be found in goral residing in lower density groups.

## Materials and Methods

### Ethics Statement

This study was conducted non-invasively, without animal handling. Fecal samples were collected from captive goral. Permission to conduct research at Omkoi Wildlife Sanctuary, a protected forest area in Thailand was granted by Department of National Parks, Wildlife and Plant Conservation (DNP) (Permit Number TS 0907.1/2501). Chiang Mai Night safari permissions were obtained from the staff veterinarian and mammal curator, who also were collaborators on the study. No permits were needed for the fecal sample collection. This study was approved by the Faculty of Veterinary Medicine Chiang Mai University Animal Care and Use Committee (FVM-ACUC) (Permit Number S22/2553).

### Seasonal Determination

There are three major seasons in Thailand: summer (February 16 – May 15), rainy (May 16 – October 15) and winter (October 16 – February 15). Information on daily temperature (°C) and rainfall (mm) during the study period at each facility was obtained from The Northern Meteorological Center, Meteorological Department, Ministry of Information and Communication Technology, Chiang Mai, Thailand [31].

### Animals and Sample Collection

**Figure 1:**
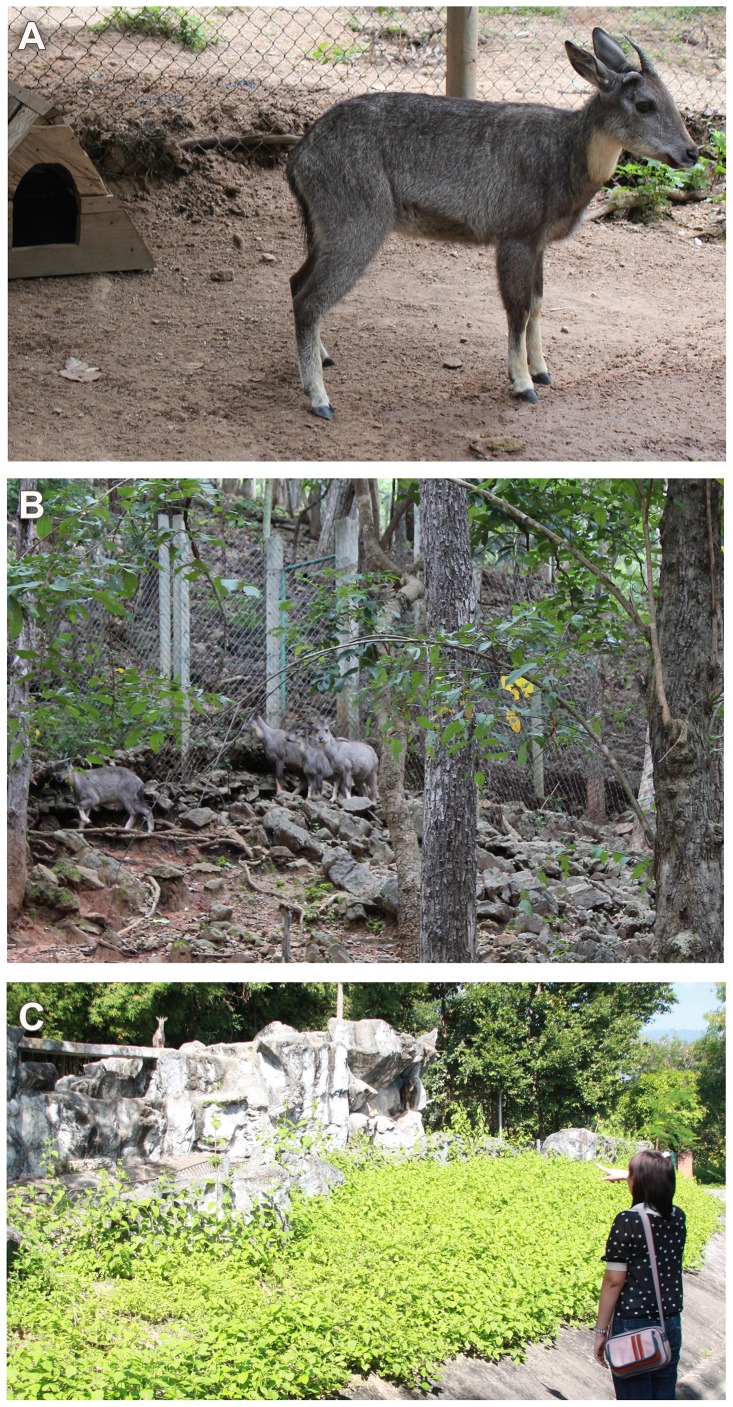
Goral pens. Examples of goral housing conditions: (a) as individuals in small pens (6 m×9 m) at Omkoi Wildlife Sanctuary; (b) in family groups in large pens (30 m×40 m) at Omkoi Wildlife Sanctuary; and (c) all animals together in a large (35 m×24 m) at Night Safari.

A total of 63 captive-born goral were used in this study; 32 were housed at Omkoi (17° 48′ 4″ N, 98° 21′ 31″ E) (n = 16 males, 16 females) and 31 at NS (18° 41′ 13″ N, 98° 55′ 8″ E) (n = 17 males, 14 females). Singleton animals were housed in 16 pens (6 m×9 m; Fig. 1A) (n = 8 males, 8 females) at Omkoi (17° 48′ 4″ N, 98° 21′ 31″ E). Another 16 goral were housed at Omkoi in larger pens (30 m×40 m; Fig. 1B), which contained one adult male, one adult female and offspring up to ∼5 months of age (8 pens; n = 8 males, 8 females; density  =  400 m^2^ per animal). At NS, 31 goral (n = 17 males, 14 females) were housed in one large enclosure (35 m×24 m; Fig. 1C) (n = 31; density  =  27 m^2^ per animal). Fecal samples from dependent offspring were not included in this study.

At Omkoi, the average age was 5.20±0.57 years for males, and 6.11±0.92 years for females. At NS, average ages were 3.43±0.59 and 4.06±1.24 years for males and females, respectively. All animals received natural light, and were fed concentrates (Betagro Company Limited, Thailand; Betagro 009 cattle finisher pellet (12% protein, 2% fat, 13% fiber, 13% moisture) and roughage (Panicum grass*; Brachiaria mutica*) once daily, with unlimited access to fresh water. A mineral block was provided in each enclosure at both facilities. All enclosures at Omkoi had dirt floors, an open shelter, a rock structure for climbing, and several natural trees for shade. The enclosure at NS contained an artificial rock and cliff structure in the middle, with about 13 m of dirt area behind it. Goral stayed primarily on the rock structure, which was ∼5 m from the public area, separated by a water mote. NS was open to the public from 1100 to 2200 hours daily.

For health care and status, there was a staff veterinarian at each facility. All animals received annual physical examinations and blood chemistry analyses. They were dewormed every 3 months at Omkoi and every 6 months at NS. Keepers were responsible for noting any changes in health status; all animals were considered in good health during the study period. Identification of feces from individuals was accomplished through keeper observations. At both facilities, old feces were removed every evening, and freshly defecated feces (∼30 g) were collected between 0830 and 0930 hours every morning from each goral 3 days/week for 1 year. All samples were stored at -20°C until processing.

### Fecal Extraction

All chemicals were obtained from the Sigma Chemical Company (St. Louis, MO) unless otherwise stated. Wet fecal samples were dried using a conventional oven at 60°C for ∼24–48 hours and stored at −20°C until extraction. Frozen dried fecal samples were thawed at room temperature, mixed well and 0.1 g (±0.01) of dry powdered feces placed in a glass tube containing 90% ethanol in distilled water. Samples were extracted twice by shaking with a Multi Pulse vortexer (Glas-Col, Terre Haute, IN) set at 70 for 30 min, centrifuging at 2500×g for 20 min and drying the combined supernatants under air in a 50°C water bath. Dried extracts were reconstituted by vortexing for 1 min in 1 ml dilution buffer (0.1 M NaPO_4_, 0.149 M NaCl, pH 7.0). The extracts were stored at −20°C until further analysis [28]. Extraction efficiency of glucocorticoid metabolites from feces was 89.2% based on the recovery of cortisol added to dried fecal samples before extraction.

### High Performance Liquid Chromatography

The numbers and relative proportions of immunoreactive glucocorticoid metabolites in goral fecal extracts were determined using reverse-phase high performance liquid chromatography (HPLC) [29]. Five fecal extracts from five gorals representing different months were combined, air dried, re-suspended in 1 ml methanol, dried again and stored at −20°C until further processing. Extract pools were reconstituted with 0.5 ml in phosphate buffer (0.01 M NaPO_4_, 0.14 M NaCl, 0.5% bovine serum albumin, pH 5.0) and filtered through a C-18 matrix cartridge (Spice™ Cartridge, VWR, West Chester, PA). The cartridge was washed with 5 ml distilled water and the total steroids eluted with 5 ml 100% methanol, evaporated to dryness, then reconstituted in 300 μl of 100% methanol containing ^3^H-cortisol and ^3^H-corticosterone (∼3,500 dpm each). Filtered fecal extracts (55 μl) were separated on a Microsorb C-18 column (Reverse Phase Microsorb™ MV 100 C18, 5 μm diameter particle size; Varian Inc., Woburn, MA) using a linear gradient of 20-100% methanol in water over 80 min (1 ml/min flow rate, 1 ml fractions). A subsample of each fraction (100 μl) was counted for radioactivity in a dual-label channel beta scintillation counter (Beckman, Fullerton, CA) to determine the retention times for the radiolabeled reference tracers. The remainder of each fraction (900 μl) was evaporated to dryness, reconstituted in 200 μl assay buffer (0.1 M NaPO_4_, 0.149 M NaCl, 0.1% bovine serum albumin, pH 7.0) and an aliquot (50 μl) analyzed in singlet in the enzyme immunoassay (EIA).

### Enzyme Immunoassay

A single-antibody cortisol EIA was used to quantify glucocorticoid metabolites, which relied on a polyclonal antibody produced in rabbits against cortisol-3-carboxymethyloximine linked to bovine serum albumin (R4866). Horseradish-peroxidase (HRP)-conjugated cortisol served as the label and cortisol was used as the standard. The cortisol R4866 antibody crossreacts with cortisol (100%), prednisolone (9.9%), prednisone (6.3%), cortisone (5.0%), corticosterone (0.7%), 21-deoxycortisone (0.5%), deoxycortisone (0.3%), 11-desoxycortisol (0.2%), progesterone (0.2%), 17α- dihydroxyprogesterone (0.2%), 17α- dihydropregnenolone (0.1%), pregnenolone (0.1%), androstenedione (0.1%), testosterone (0.1%), androsterone (0.1%), dehydroepiandrosterone (0.1%), dehydroisoandrosterone-3-sulfate (0.1%), aldosterone (0.1%), estradiol-17β (0.1%), estrone (0.1%), estriol (0.1%), spironolactone (0.1%) and cholesterol (0.1%) [30]. The EIA was performed in 96-well plates (Nunc Maxisorp, Fisher Scientific, Pittsburgh, PA) coated 16–24 hours previously with cortisol antiserum (50 μl in coating buffer, 0.05 M NaHCO_3_, pH 9.6; 1∶10,000 dilution). Cortisol standards (50 μl, range 3.9–1000 pg/well), diluted in assay buffer and samples (50 μl, 1∶2 dilution) were combined with cortisol-HRP (50 μl; 1∶15,000 dilution) and incubated at room temperature for 1 hour. Plates were washed five times (Biochrom Anthos Fluido 2 microplate washer, Cambridge, UK) before addition of 100 μl substrate (0.4 mM ABTS) to each well. After incubation for 15–30 min, the absorbance was measured at 405 nM (TECAN Sunrise microplate reader, Salzburg, Austria) until the optical density approached 1.0. The cortisol antibody and HRP were obtained from Coralie Munro (University of California, Davis, CA, USA).

The assay was validated for goral feces by showing that serial dilutions of pooled extracts produced displacement curves parallel to those of the cortisol standard curve. Pearson's correlation coefficient analyses were used to determine the correlation in percent binding between serial dilutions of hormone standards and fecal extract dilutions in the parallelism validation tests (r = 0.9595). Addition of unlabeled cortisol standard (Sigma Diagnostics Cat. #H4001) to pooled fecal extracts before extraction resulted in a significant (*p*<0.05) recovery of mass for female (y = 1.03×–0.10, R^2^ = 0.99) and male (y = 0.97×–0.14, R^2^ = 0.99) goral. Physiological validation of the cortisol EIA was demonstrated by showing a significant increase (100–150% increase; *p*<0.05) in concentrations within 24–48 hours after a stressful event (e.g., blood collection, n = 2; semen collection, n = 4). Assay sensitivity was 0.078 ng/ml at 90% binding. Inter-assay CVs were <15% based on binding of high (30%) and low (70%) control samples. Samples were re-analyzed if the duplicate CV was >10%; thus, intra-assay CVs were <10%. Data are expressed as ng/g dry feces.

### Data Analysis

Sixteen goral at Omkoi housed in individual pens (n = 8 males, 8 females) were used to study the effect of gender and season on glucocorticoid excretion. Sixteen goral in eight large pens at Omkoi (n = 8 males, 8 females) and all adult goral at NS (n = 17 males, 14 females) in one large pen were used to study the effect of management at each facility on glucocorticoid production. Goral at NS also were used for the seasonal analysis. Data are reported as the mean ± standard error of mean (SEM). Glucocorticoid metabolite concentrations were averaged by week, followed by calculations of seasonal means. Data were analyzed by fitting a linear model using Generalized Least Squares method with R version 3.0.0 [32] and *nlme* package 3.1-110 [33]. Differences across gender (male vs. female), season (summer vs. rainy vs. winter) and animal density (large at Omkoi vs. large at NS) were analyzed using GLS for repeated measures data followed by a Bonferroni test for multiple comparison analysis. The significance level (α) was set at 0.05.

## Results

**Figure 2:**
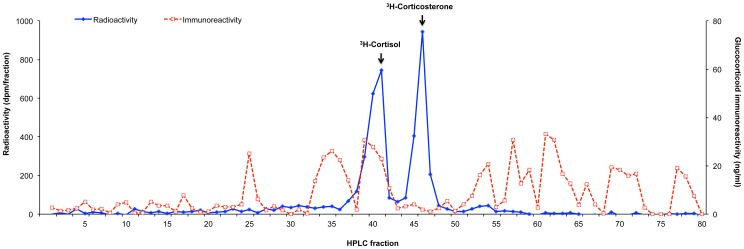
Chromatographic analysis of glucocorticoid metabolite immunoreactivity. Immunoreactivity of glucocorticoid metabolites in fecal extracts of goral was determined by reverse-phase HPLC analysis. Glucocorticoid concentration in each fraction was determined using a cortisol EIA. Elution of ^3^H-cortisol and ^3^H-corticosterone reference tracers in HPLC fractions of extracted fecal samples are indicated by the arrows.

Analysis of HPLC-purified fecal eluates from male goral revealed the presence of several glucocorticoid metabolites, one of which co-eluted with the cortisol tracer (fraction 39–42) and represented 17.5% of the immunoreactivity (Fig. 2). Two immunoreactive peaks at fractions 25 (4.7%) and 33–37 (18.8%) appeared to be more polar, and five peaks (58.9% total immunoreactivity) were less polar than the tritiated reference tracers.

**Figure 3:**
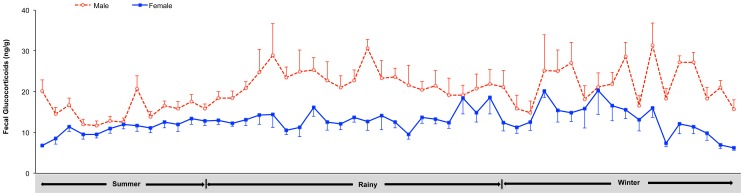
Seasonal pattern of fecal glucocorticoids. Longitudinal mean (± SEM) fecal glucocorticoid metabolite concentrations for male and female gorals were determined by a cortisol EIA. Fecal samples were collected from February 2010 through February 2011, representing the summer (February 16 – May 15), rainy (May 16 – October 15) and winter (October 16 – February 15) seasons.

There was no difference in age between males and females at the two facilities. Fecal glucocorticoid metabolite concentrations were consistently higher (*p*<0.05) in male than female goral at Omkoi (Table 1, Fig. 3). For both sexes, mean glucocorticoid metabolite concentrations differed across seasons and were higher in the rainy season and winter, and lower in the summer (*p*<0.05) (Table 1, Fig. 3). There was no difference in glucocorticoid metabolites between the rainy season and winter for either sex (*p*>0.05) (Table 1).

**Table 1:** Mean (± SEM) fecal glucocorticoid metabolite concentrations (ng/g) between male and female goral at Omkoi Wildlife Sanctuary across the three seasons in Thailand.

Season	Male	Female
Summer	15.30±0.49^a,1^	11.22±0.37^b,1^
Rainy	22.10±0.73^a,2^	13.37±0.44^b,2^
Winter	21.98±0.98^a,2^	13.27±0.71^b,2^

a,b Values differ between male and female gorals, different letters indicate differences (*p*<0.05).

1,2 Values differ among seasons, different numbers indicate differences within the same gender

(*p*<0.05).

Data on seasonal average daily temperature and rainfall between Omkoi and NS are shown in Table 2. Average temperature at NS was higher than that at Omkoi in every season (*p*<0.05). Temperatures differed across season for both facilities with the same trend, and were highest during the rainy season and lowest in the winter (*p*<0.05), although the difference between summer and rainy seasons at NS was not significant. The amount of rainfall was similar across facilities for summer and winter (*p*>0.05), but was significantly higher at NS compared to Omkoi. At each facility, rainfall was highest in the rainy season, intermediate in the summer and lowest in the winter (*p*<0.05).

**Table 2:** Seasonal mean (± SEM) average daily temperature and rainfall at two captive goral facilities in Thailand.

Environmental data	Season	Omkoi	Night Safari
Average temperature (°C)	Summer	23.47±2.30^a,1^	27.88±2.73^b,1^
	Rainy	24.59±1.85^a,2^	28.21±2.13^b,1^
	Winter	19.77±1.69^a,3^	23.88±2.05^b,2^
Rainfall (mm)	Summer	2.92±0.29^a,1^	2.34±0.22^a,1^
	Rainy	5.19±0.39^a,2^	6.39±0.48^b,2^
	Winter	1.40±0.12^a,3^	1.14±0.10^a,3^

a,b Values differ among facilities, different letters indicate differences (*p*<0.05).

1,2 Value differ among seasons, different numbers indicate differences within the same facility (*p*<0.05).

**Figure 4:**
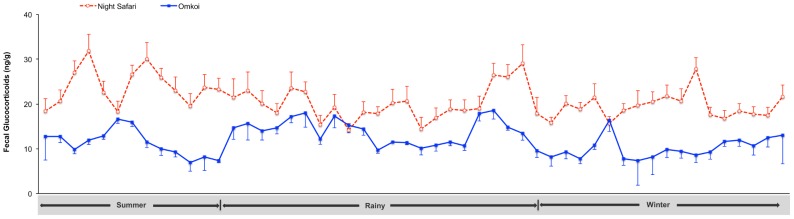
Effect of housing on glucocorticoid production. Longitudinal mean (± SEM) fecal glucocorticoid metabolite concentrations for goral housed at two facilities in Thailand, determined by a cortisol EIA. Fecal samples were collected from February 2010 through February 2011, representing the summer (February 16 – May 15), rainy (May 16 – October 15) and winter (October 16 – February 15) seasons.

A comparison of glucocorticoid metabolite concentrations between Omkoi and NS is shown in Table 3. Further seasonal analyses revealed a significant facility effect on glucocorticoid concentrations in the summer, being lowest at Omkoi and highest at NS (Table 3, Fig. 4). Within Omkoi, glucocorticoids were lower in the summer (*p*<0.05), with concentrations being similar between the rainy season and winter (*p*>0.05). By contrast, at NS, the highest concentration was observed in summer (*p*<0.05), again with rainy and winter seasons being similar.

**Table 3:** Facility effect on mean (± SEM) fecal glucocorticoid metabolite concentrations (ng/g) in goral housed in large (30 m×40 m, area 400 m^2^ per animal) enclosures at Omkoi Wildlife Sanctuary, and one large enclosure (35 m×24 m, area 27 m^2^ per animal) at Night Safari across the three seasons in Thailand.

Season	Omkoi	Night Safari
Summer	9.94±0.60^a,1^	23.89±0.83^b,1^
Rainy	12.54±0.43^a,2^	20.08±0.64^b,2^
Winter	12.76±0.67^a,2^	19.45±0.54^b,2^

a,b Values differ among enclosure sizes, different letters indicate differences (*p*<0.05).

1,2 Values differ among seasons, different numbers indicate differences within the same enclosure sizes (*p*<0.05).

Across facilities, animals exhibited significantly higher glucocorticoid metabolite concentrations at NS compared to Omkoi (Table 3, Fig. 4). Because of the within facility difference in summer glucocorticoid responses (being lower at Omkoi and higher at NS), the overall difference between facilities was more than double in that season. By comparison, concentrations at NS were only about a third higher in the rainy and winter seasons compared to Omkoi. Moreover, goral at NS had a lower area per animal; the stocking density at NS was about 14 times greater than that at Omkoi.

## Discussion

A cortisol EIA was validated for quantifying fecal glucocorticoids in goral, a threatened species of national importance in Thailand. Findings support the hypothesis that glucocorticoid concentrations are higher in goral housed in animals maintained as one large group at NS compared to smaller, breeding groups at Omkoi. There also was a seasonal effect on glucocorticoid production, although it differed by facility. Specifically, in the summer, concentrations were lowest at Omkoi and highest at NS, possibly due to environmental differences. Higher fecal glucocorticoid metabolite concentrations were observed in male than female goral, irrespective of facility and season. Thus, results suggest that glucocorticoid production in goral is influenced by physiological, environmental and captive conditions, several of which have welfare and management implications.

HPLC analysis found the majority of glucocorticoid immunoreactivity in goral fecal extracts was associated with several peaks, two of which were more polar and five that were less polar than the tritiated reference tracers, indicating the presence of multiple metabolites. A proportion (17.5%) of the immunoreactivity co-eluted with radiolabeled cortisol. Several radiometabolism studies have demonstrated the near absence of authentic radiolabeled cortisol and corticosterone in feces; for example, in carnivores [34], [35], lagomorphs [36], domestic livestock [37], [38] and primates [39]. By contrast, immunoreactive substances in feces of the Himalayan black bear (*Ursus thibetanus*) and clouded leopard (*Neofelis negulosa*) co-eluted with ^3^H-cortisol, suggesting these species may excrete native cortisol in variable amounts [29], and so it appears that goral do as well.

Gender differences in adrenal activity have been reported previously and may be related to a variety of physiological and behavioral changes within each sex [23]. As with goral, other studies have shown that levels of glucocorticoids (serum or fecal) are higher in males than females, including the laboratory rat [40], marmoset (*Callithrix jacchus*) [41] and spider monkey (*Ateles geoffroyi yucatanensis*) [42]. By contrast, a number of studies have reported higher concentration in females, such as in the domestic dog and cat [35], clouded leopard (*Neofelis nebulosa*) [12], sheep [43], [44] and chimpanzee (*Pan troglodytes*) [45]. Still others report no difference between genders; e.g., red deer (*Cervus elaphus*) [13], black (*Diceros bicornis*) and white (*Ceratotherium simum*) rhinoceros [46] and reindeer (*Rangifer tarandus*) [47]. It is not clear if such gender differences are strictly species dependent, or are influenced by other physiological factors. It has been suggested that when glucocorticoids are higher in females, differences may be related to evolutionary adaptations that increase alertness (i.e., increased anticipation of “fight or flight”) for protecting and rearing young [45], [48] or to avoid aggression from dominant males [43], particularly in species where males are larger and more aggressive [12]. Gender effects may also be due to differences in steroid biosynthesis or metabolism [49]. For example, female rats excrete less hormone into feces presumably because of higher plasma corticosterone-binding capacity [50]–[51]. There is no size difference between male and female goral, and little aggression is observed within family units. Similarly, infanticide is rare in this species. In the wild, gorals are polygynous and dominant males defend territories and access to females during the breeding season through threatening displays and combat with other males [3]. Thus, males might maintain higher levels of glucocorticoids on average to generate an advantage over competing males, a strategy that persists in captivity.

Glucocorticoid metabolite concentrations in goral varied across seasons, and overall means for both males and females were higher during the rainy season and winter than in the summer. Increased production of glucocorticoids enhances catabolic function during the winter as an adaptation to cold weather [13]. Other seasonal species show fluctuations in fecal glucocorticoid levels related to climate and/or the breeding season. A study of red deer showed a marked increase in fecal glucocorticoid metabolites in December and January, which followed the breeding season in September through November [13]. Deer mice (*Peromyscus maniculatus*) and red-backed voles (*Clethrionomys gapperi*) exhibit increases in fecal glucocorticoids in late August to late September and in mid- to late September, respectively, again following the late summer, early fall breeding seasons [52]. In free-ranging male muriqui monkeys (*Brachyteles arachnoides*), fecal glucocorticoid concentrations are increased during the mating period, which corresponds to the dry season in Brazil [53]. And in African elephants (*Loxodonta africana*), fecal glucocorticoids are higher in the dry season, presumably because of reductions in natural resources [54]. The seasonal increase in glucocorticoid production in goral preceded the purported winter breeding season by several months. Food and water resources were consistent throughout the year for captive goral, eliminating that as a controlling factor. Nor was there a relationship between glucocorticoids and rainfall. However, further analyses revealed a significant facility effect with respect to seasonal glucocorticoid production, especially in the summer months, with concentrations at NS being about double those of Omkoi. Thus, we considered possible explanations for glucocorticoid differences due to both season and location. Animals at Omkoi had more shelter from the sun in the form of natural trees and a shed in each enclosure, whereas animals at NS had no such shade and the overall daily temperatures were higher. As a result, higher glucocorticoids during the summer months at NS could reflect a form of heat stress. High ambient temperatures, direct and indirect solar radiation, and humidity all are environmental stressors that affect animal welfare and can stimulate increased glucocorticoid production, as discussed for various livestock species [55]. A high Temperature-Humidity Index during the rainy season also has been suggested to be a source of stress in tropical species through alterations in the hypothalamo-pituitary-gonadal axis [56]. Secretion of cortisol stimulates physiological changes that allow animals to better cope with a hot environment [57], and for domestic cattle in South Africa, providing shade maintained lower serum cortisol concentrations and rectal temperatures [58]. Thus, the reduced fecal glucocorticoid concentrations across seasons at Omkoi, and especially in the summer, could be the result of a slightly cooler climate and perhaps more importantly, adequate shade being provided to the animals compared to NS. Moreover, Omkoi is located inside a wildlife sanctuary where wild goral live, so animals were exposed to more typical forest cover within their enclosures and a more natural climate.

Besides climate, animals are subjected to a number of other potential factors in the captive environment that can induce stress, such as health problems, limited space, artificial habitats, noise, exposure to the public and unnatural social groupings [15], [59], [60], [61]. For most species, captive facilities are not likely to match the amount of space available to free-ranging individuals, but proper husbandry can enhance welfare and the likelihood for more natural behavior [10], [12], [14], [18]. At both facilities, animals were found to be in good health by staff veterinarians, so that did not appear to be a significant factor in this study. However, the management of gorals at both facilities was different in that at NS animal density was 14 times higher than that at Omkoi. As recently reviewed by Creel et al. (2013), population density is one of the best-documented factors that influences the HPA axis. As far back as the 1950's it was recognized that increased population densities of wild and captive-held species, including mammals, birds, reptiles and amphibians, can result in antagonistic social interactions, suppression of reproduction, increased mortality and heightened adrenal activity (see review [62]). This may be particularly true for territorial species, where conspecific intrusion increases antagonistic encounters. For example, in a study of Peré David deer (*Elaphurus davidianus*), higher fecal glucocorticoids and increased aggression were observed in animals kept at a higher density [17]. Goral family units in the wild generally are under a dozen individuals, and usually include only one male for several females. Thus, a single enclosure containing 31 goral of equivalent gender numbers, such as that at NS, may be perceived as a stressor, and as a result, cause increased adrenal activity. During the breeding season, male goral can become aggressive. Based on keeper records, there was more fighting among the large number of conspecifics, especially males, at NS. By contrast, little aggression was observed among the animals housed in family units at Omkoi. Thus, limited space experienced by goral at NS could be one variable that explains the higher glucocorticoids found at this facility.

Besides more limited space, the animals at NS also were exposed to more noise and the physical presence of humans, which is a zoo and has a high rate of tourist activity. By comparison, Omkoi is a breeding center located in a wildlife sanctuary and not open to the public. The ability of zoo animals to tolerate large numbers of visitors may be species specific; some do well while others do not [63]. However, there are numerous examples of captive-held wildlife being negatively impacted by public exposure. For example, clouded leopards expressed higher fecal glucocorticoids when on display than off [12], and in spider monkey (*Ateles geoffroyii rufiventris*), the number of visitors had a stimulatory effect on the hypothalamic–pituitary–adrenal (HPA) axis [64]. Zoo visitor density also increased fecal glucocorticoid excretion and aggressive behavior in blackbuck (*Antilope cervicapra*) [65], whereas in black rhino, fecal glucocorticoids and mortality rates were correlated positively with the percentage of public visitor access around the enclosure [26], and in honeycreepers heightened glucocorticoid excretion was observed in animals exposed to environmental disturbances caused by humans and equipment [14]. Thus, forced proximity to humans can be harmful to animal well being in captive situations [15]. At NS, not only were goral exposed to tourists for 11 hours per day, but also the public area was only about 5 meters away from the animals. There was only a narrow water mote separating the two, with a rock structure abutting the mote. Goral spent most of the day on the rocks, and so were quite close to the visitors. As reviewed by Tarlow and Blumstein (2007), the distance by which an animal begins to flee from an approaching human is known as the ‘flight-initiation distance’ (FID), and can be used to define ‘set-back distances’ or ‘buffer zones’ when designing facilities. Goral at NS may perceive visitors as being too close, and this could be having an impact on chronic adrenal activity. An analysis of FID at NS could determine if the amount of set-back between goral and the public is adequate [66].

## Conclusion

It is undeniable that non-invasive fecal glucocorticoid metabolites monitoring is a valuable tool for advancing our understanding of adrenal function and stress responses in wildlife, and can enhance the *ex situ* management of threatened species. This study validated an EIA for assessing fecal glucocorticoids in goral, a species of high priority in Thailand, and found higher concentrations in males than females, and in animals housed at a higher animal density and exposed to human visitors. As designed, it was not possible to discriminate between the stress caused by a higher animal density or public exposure in this study; there were confounding factors at NS. Thus, additional studies are planned to determine with more certainty what factor(s), exposure to the public, area per animal or stocking density, impact individual animal welfare the most. Nevertheless, we have identified several potential stressors pertaining to the welfare of captive goral. Additionally, we will be relating fecal glucocorticoid metabolites measures with those of reproductive hormone metabolites in the same samples to help unravel how “stress” may be modulating reproductive function/performance and/or success. Together, this information will be crucial for guiding efforts to improve management and create self-sustaining and healthy populations of this nationally important species.
